# Proceedings of Veterinary Societies

**Published:** 1888-10

**Authors:** 


### PROCEEDINGS OF VETERINARY SOCIETIES.

United States Veterinary Medical Association—Twenty-fifth Annual Meeting.—The Comitia Minora of the United States Veterinary Medical Association met at the Rossmore Hotel, New York city, at 9.45 a. m. on September 18, 1888, and at 10.30 a. m. reported to the general meeting, at which President Huidekoper presided.

The roll call of the twenty-fifth annual meeting was answered by forty-three members. After the approval of the minutes of the previous meeting, the Comitia Minora recommended for membership all applicants for membership who were graduates, except Dr. Ward of Baltimore, whose application was refused on the grounds of unprofessional conduct, in his methods of advertising. The name of Edgar R. Martin, of Philadelphia, was laid over until the next meeting, on the ground of similar charges. Dr. Middleton, a non-graduate, was admitted to membership. The Association approved the recommendation of the Committee to expel from membership Drs. Spranklin, of Baltimore, and Jones, of Long Island, for violations of the code of ethics.

Dr. Coates, Chairman of Committee on Intelligence and Education, reported at some length on recent legislation in New York State in the interest of veterinarians; referred to the death of Crowley at Central Park from tuberculosis, and led on to the great importance of the recent Congress at Paris, called to consider the great dangers of its transmissibility from animals to man through the ingestion of meat and milk. Noted the establishment of Chairs of Veterinary Science at Quebec, Canada and at the State College in South Carolina.

Dr. Hoskins, Chairman of Special Committee to procure uniform standard for different schools of Veterinary Science, announced another addition to the three term schools.

Dr. Zuill, Chairman of Committee on Infectious and Contagious Diseases, commented upon the prevalance of contagious pluro pneumonia and tuberculosis, referring very forcibly to the dire need of legislative action, relative to the checking of the progress of the latter disease. Cited the dangerous extent of glanders and farcy in certain localities, particularly parts of Pennsylvania. Furnished statistics of the extent of anthrax, rabies, and hog cholera. Extolled the beneficent work of Dr. Salmon and the able corps in the Bureau of Animal Industry.

Dr. Pendry, Chairman of Committee on Army Legislation, reported the slow progress of the bill. He was followed by remarks from Dr Huidekoper who advised the recall of the present bill and its need of revision.

An election of officers for the ensuing year then took place resulting as follows: President, Dr. R. S. Huidekoper, Philadelphia, Pa.; Vice-President, Dr. D. J. Dixon, Hoboken, N. J.; Secretary, Dr. W. Horace Hoskins, Philadelphia, Pa.; Treasurer, Dr. Jas. L. Robertson, New York City; Comitia Minora: Drs. Zuill, McLean, Clements, Ross, Howard, Winchester and May.

A rising vote of thanks was unanimously tendered the retiring secretary, Dr. Chas. B. Michener, for his zealous and effic ent services.

Dr. R. S. Huidekoper read a very carefully prepared and highly interesting paper on the "Origin of the Domestication of the Horse."

The subject of Tuberculosis was then taken up, and, after considerab'e discussion, a committee composed of Drs. Liautard, McLean and Clements were named to draft a series of resolutions, relative to the rapid increase, the dangers from inges'ion of meat and milk of tuberculosis animals, the need of properly qualified veterinarians as inspectors of dairies, markets and slaughter houses, instructing them to forward these resolutions to the Medical Congress in session at Washington, D. C., and a copy to all City, State and National Boards of Health.

Dr. J. E. Myers, of Cincinnati, then referred to a recent article describing a disease known as "Mad Itch," which had been written up by him about the years 1869 to 1871, during an enzootic outbreak in a distillery stable, used for fattening purposes.

After a vote of thanks to the assayists, the meeting adjourned to meet at Boston the coming March.

In the evening a banquet was given at the Rossmore Hotel, where several toasts were happily responded to by some of the invited guests and members.

W. HORACE HOSKINS, Secretary,

12 S. 37th St., Philadelphia, Pa.

THE NEW JERSEY STATE VETERINARY SOCIETY.—The annual meeting of this Society was held on Saturday, August 4th, 1888, at Hotel Shelburne, Long Branch, N. J., Dr. J. C. Corlies, President in the chair.

The Secretary called the roll, which was answered by a fair number of veterinarians from various parts of the State.

The President read his annual address in which he spoke of the past, present and future of the veterinary profession in New Jersey, at the end of which he was loudly applauded.

The annual election of officers resulted as follows:

Dr. J. C. Corlies, of Newark, re-elected President.

Dr. Theodore De Clyne, of New Durham, first Vice-President.

Dr. Eldon L. Loblein, of New Brunswick, second Vice-President.

Dr. W. H. Lowe was proposed as Secretary, but he refused to be a candidate, as he had held the office for a number of years, and so had his name withdrawn in favor of Dr. Charles Keuhne of Jersey City, who was elected to that office.

Dr. Joseph F. Autenreith, of Jersey City, Treasurer.

Dr. Andrew Sherk of Newark, Dr. Joseph Naylor of Jersey City, Dr. Elmore R. Mercer of Montclair, and Dr. Matthew A. Pierce of Patterson, were re-elected as the Board of Censors.

Dr. J. S. Sutcliffe of Middletown, N. Y., read a paper on Hernia and also one on Pneumonia, and spoke of several interesting cases which came under his observation. A vote of thanks was tendered the doctor for the interesting paper.

The Society adjourned to meet at Jersey City, November 1st, 1888.

CHARLES KUEHNE, Ph. G., D.V.S., Secretary.

THE OHIO STATE VETERINARY MEDICAL ASSOCIATION held its semi-annual meeting in the Music Verein Hall at Cincinnati, July 26th, 1888. The meeting was called to order by the President, Dr. J. S. Butler, who made a few appropriate remarks.

Twenty-five members answered to roll call.

The name of E. H. Shepard was proposed for membership, and being a graduate he was unanimously accepted.

Dr. Derr, of Sidney, then read an essay on Azoturia, and Dr. J. C. Meyer, Sr., one on Antifebrin. Dr. J. D. Fair read a paper reporting some cases of Pleuro-pneumonia in horses, which came under his observation. Dr. J S. Butler referred to a case of Tuberculosis in an eleven months old calf, whose lungs weighed thirty-two pounds; also to an enlarged spleen of a horse, the organ weighing fifty-five pounds. Dr. Meyer, Sr., referred to a similar case in which the spleen weighed seventy-nine pounds. Dr. J. S. Butler also reported the result of his investigation of *maladie du coit* in Illinois.

The above subjects were well discussed, most of those present taking part in the discussion.

The constition and by-laws were so revised that none but graduates from a recognized college can be admitted into the Association. It was also arranged that the annual meeting hereafter shall be held at Columbus. Drs. Derr and Meyer were requested by the Association to send a copy of their papers to the *Veterinary Review* and JOURNAL OF COMPARATIVE MEDICINE AND SURGERY for publication, which they consented to do.

The meeting then adjourned. G. W. BUTLER, Corresponding Secretary.

BRITISH MEDICAL ASSOCIATION.—TRANSMISSION OF DISEASE FROM ANIMALS TO MAN.—Professor Walley said that anthrax stood in the fore-front of all maladies of the class which had to be discussed. He knew it was not the disease which was at the present moment occupying the minds and attention of sanitarians, but it was a most deadly one, and it was rapidly communicated from animals to man, and that too in a variety of ways. Bearing upon anthrax there was a certain amount of legislation, and therefore the disease had so far passed out of debateable ground. The regulations, however, were deficient in respect that they were to a very large extent permissive, and not uniform over the whole country. In 1881 seventy dogs and two horses in Edinburgh came under his personal observation as suffering from rabies. No action was taken by the Corporation until two human lives were lost, and then the advice was taken that had been previously profferred, and the result was that in less than three months the disease was stamped out. If that disease could be stamped out of Edinburgh in three months, it could be stamped out of the whole country in from six to twelve months. (Applause.) Rabies was now also included in the Contageous Diseases (Animals) Act, and could therefor be said to have passed out of the realm of discussion to an extent. The malady which at present occupied the forefront in this connection was tuberculosis. That disease ought to have been recognized as communicable from animals to man many years ago; and it should be dealt with as one of the greatest pests to humanity, and also as one of the greatest pests to the animal creation. He had not the slightest hesitation in saying that it was communicable from animals to man, and back again from man to animals in every possible shape and form. It was communicable without any doubt by ingestion, by inhalation, and by direct inoculation, and probably in other ways that they knew little of. There were probably more deaths produced by this disease than all the zymotic diseases put together. When he told them that out of thirteen animals that were slaughtered in the Edinburgh slaughter-house some two or three months ago on account of pleuro-pneumonia, there were no fewer than six affected with tuberculosis, two of which were as bad cases as he had ever seen, they would have some notion as to the extent of the disease amongst animals. The disease was simply slaughtering poultry by thousands in all parts of the country, and it seemed to be a remarkable fact that, despite the high temperature of birds, it ran a very much more rapid course in poultry than it did in animals and killed them with more certainty. In his opinion the disease might be transmitted by eggs. If there was not any actual proof there was a vast amount of indirect proof of the transmission of disease from animals to man. There was now accumulated a very large amount of evidence in this direction, that the children at least, if not grown up persons, might, and did contract the disease from animals by ingestion of milk. There was practically no direct evidence of the transmission of tuberculosis from animals to man by the medium of flesh, but here again there was a vast extent of indirect evidence. The milk, he did not think, could be affected where the udder of the animal was not affected by the disease. Principal Walley concluded by moving "That the public health section of the British Medical Association approves of the action of the Government in inquiring by commission into the relationships of bovine and human tuberculosis, and urges that as soon as possible such measures be taken as the report of the commission might show to be advisable; that copies of this resolution be forwarded to the Prime Minister, the Secretary for Scotland and the Lord Advocate." (Applause.) He remarked that the Magistrates of Edinburgh were the only body that had taken from the public market or from the byre animals suffering from tuberculosis, and paid compensation for them. This they had done on the representations of their veterinary adviser.

Dr. Farquharson, M. P., said that scarlet fever could be transmitted from the cow, but he thought that they would agree with the lecturer that all such diseases fell into insignificance before that terrible disease tuberculosis, which, he thought, they would all see from what they had heard could be communicated from animals to man. He thought they should not rest until they had this disease included among those other diseases of animals which were mentioned in the Contagious Diseases (Animals) Act. Of course the communicability of the disease of animals to man was raised to an acute point now, because it was now established that the bacilli of the human tuberculosis and the animal tuberculosis were the same. The prospect was really a terrible one. If this disease went on increasing in the cow and our people got poorer every year, he was afraid that the terrible disease of consumption would become more and more rife. Milk has been shown to be often swarming with bacilli, and now even the safe and simple egg, which they had thought safe because buttressed round by shell, was to be taken away from them. After the evidence that had been brought forward of the communicability of this disease and the great variety of sources through which it might be communicated, he thought the adoption of some such resolution as that which had been proposed became necessary.

Prof. Edgar Crookshank, King's College, London, pointed out that it had been known from the earliest times that certain diseases of the lower animals were comumnicable to man. In later years it had been shown that certain diseases such as tuberculosis were communicable to the lower animals, but with the exception of hydrophobia insufficient attention had been paid to the diseases common to man and animals. Jenner was the first to draw attention to the diseases of the cow's udder. In his inquiry on cowpox Jenner led the way to the study of these diseases. The diseases under discussion might be divided in the present state of their knowledge into three classes. In the first might be placed such diseases as cowpox, foot-and-mouth disease, anthrax and glanders which never occurred in man independently of the disease in the lower animal In the next class might be placed tuberculosis and actinmycosis, which possibly were communicated from the cow. He had heard it stated that the origin of the specific poison of human infectious fevers was a new and startling theory; it was startling, but by no means new. The Arabians believed that smallpox originated from a disease of the camel, while Jenner believed that it arose from grease of the horse, and Henry Jenner maintained that cowpox was smallpox.

Dr. Buist, Edinburg, said that Dr. Fleming, who should have opened the discussion, took up the position that the human and animal variety were specifically distinct diseases, and he did so on the ground that smallpox could not be communicated from man to the cow, although it might be communicated from man to the horse, and he said that on that account he considered that cowpox and smallpox were specifically distinct. He thought that the decision of this question of identity would be best settled by a study of the natural infective material and its effects. If this was difficult in such diseases as smallpox and cowpox where the infective materials had definite effects, it was much more difficult in cases such as scarletina, where the nature and the effects of the infective material were indefinite. Infective materials were natural and cultivated, and the effects of these were very different. While in cow-pox and variola the natural material produced local effects, cultivated materials produced no local effects but eruptions. This was seen in calves and monkeys; cultivated variola producing an eruption in the latter on the fifth day or in half the time required by the natural material. The true infective material in vaccine and variolous lymph capable of reproducing the same disease was suspended in it in the embryonic condition. This was the bacteric form existing in these infective materials. Where the infective material was composed of bacilli, as in anthrax and tubercle, it was comparatively easy to trace disease from animals to man, but where their form was very minute and indefinite it was much more difficult. In illustration of this he referred to the opposing views of Dr. Klein and Professor Crookshank with regard to the Hendon cow disease and the Wiltshire cow disease. He did not think that in either case was the source of the material used for pure cultivation free from objection, because it was derived from the discharge and tissue of the ulcers instead of from the early crusts in which the infective material of both cow diseases would have most certainly been found. No definite conclusions could therefore be come to as to the nature of the cultivated infective materials in either.

Dr. Seaton, St. Thomas' Hospital, London, said it was a recognized fact that disease in the cow was related in some way or another to the disease that we called scarlet fever. (Cries of "No, No," and applause.) There was direct evidence he contended, as to the possibility of milk communicating scarlet fever. That evidence rested upon the observation of some of the most accurate observers. The number of cases of scarlet fever attributed to milk had, of late years, been getting very large. These cases might be divided into three classes—First of all there were the cases in which milk had been admitted to be the cause of the disease; that was where the distribution of the disease coincided with the distribution of the milk, and there was some evidence that it might have been contaminated by human agency. Such an outbreak as that at Newcastle might be cited as an example of this kind. The second class of cases were those in which medical officers were wholly unable to find any sources of human contamination. In these cases no observations might have been made at all as to the possibility of the existence of disease among cows. Then came the third class of cases in which there was undoubtedly a distribution of a disease coinciding with milk—the milk, that was to say, was the cause of the disease, no evidence of human contamination being discoverable—and at the same time there was disease in the udder of the cow. Under this last head there were cases of which it was not enough to say that some human source of infection must have been overlooked. The evidence was very strong upon the point that there was no possibility of human infection, and in such cases it became the business of veterinarians to closely study the disease which was possibly the source of the specific infection.

Dr. Edington, Edinburgh, stated that he felt inclined to take exception to the remark of Dr. Farquharson that scarlet fever had been shown to be due to a disease in the cow. He held that there was not evidence, either pathological or otherwise, to show that such a hypothesis was at all tenable. He then referred to previous researches he had made and stated that he had found the microcosms which he had before found in scarlet fever, and which the Edinburg Medico-Chirurgical Society held were the same as Dr. Klein found in vaccine lymph. He did not consider that such an organism was in any way specifically related to the vaccinia. He thought it was only a contamination. He then showed that Professor Park of New York, and Dr. Stickler of New Jersey, had already corroberated the prescence of his (Dr. Edington's) baccillus in scarlet fever, and the latter of whom had gone further and produced in animals the same results as himself.

Professor M'Fadyean, Edinburgh, said that a very natural, though somewhat rough division of diseases that were communicable to man might be made into two classes—those that were due to macro-parasites or animal parasites; and those that were due to micro-parasites or vegetable parasites. Professor Walley had said that he would be inclined to place anthrax in the first position as a disorder communicable both to man and to animals. He (the speaker) thought that in one sense that was true, but in another sense it required to be stated with some reservation. Anthrax was common in the lower animals, but not very common in man. He had been anxious to get the history of cases of anthrax communicated to human beings by means of anthrax flesh, but he had not succeeded. That was not because anthrax flesh was not consumed, because wherever anthrax broke out the flesh was certain to be sold in the ordinary course and consumed. A previous speaker had said that he considered the fact that a disorder prevailed among cows that was capable of exciting scarlatina among human beings had been established. As one who had impartially followed both the direct and the indirect evidence that had been produced in this country within the last few years on the subject, he demurred to that statement. If he followed that speaker correctly, he said that where they had investigations conducted by medical officers of integrity and ability, who failed to discover any human contamination in the milk, they were almost forced to conclude that it had an animal source. Professor M'Fadyean maintained that there was not very much force in the reasoning, and he stated that he had had sent to him a report from the medical officer of health for Newcastle giving details of an outbreak of scarlatina there which showed that the officer had been unable to trace any connection between the milk supply and the outbreak of scarlatina. With regard to tuberculosis, he had worked at the disorder for a number of years and he could certainly state, as the result of his obvervations, that tuberculosis was an extremely common disorder in cattle. He had taken measures to ascertain the prevalence of the disorder; and although in the districts in Scotland in which it was most prevalent it caused annually the loss of from five to ten per cent. in dairy cattle, he was shocked to learn that in some districts there was a proportion of fifty per cent., and even eighty and ninety per cent. of animals killed for tuberculosis. He could hardly believe that statement. He thought there must be an error somewhere, because it was the fact that in other countries young animals had a considerable immunity from tuberculosis. He believed that the identity of human and bovine tuberculosis had been clearly established, though they had not direct evidence or proof that human beings might be infected from a bovine source, they had as nearly as possible such evidence. They had not complete evidence because they could not take a human being and [illegible] im with tubercular milk or meat.

Professor M'Call, Glasgow, expressed his agreement generally with Professor Walley, but said the only point upon which he was not so clear was that tuberculosis might be propagated in the egg; in other words, he doubted if the organism was contained in the egg. In his own practice he had never seen any reason to corroborate that statement, but it did not follow that he was perfectly correct. Professor Walley had led them to understand that unless the udder of the cow was locally affected, tuberculosis would not be transmitted. He (the speaker) differed very much from that view. Experiments had shown that the milk of an animal infected with tuberculosis, and whose udder was perfectly healthy, had transmitted the disease. That had been seen in more than one instance. He did not think Professor Walley had given them to understand that tuberculosis was an extremely prevalent disorder. Other speakers had said that about fifty per cent., and one speaker had declared that eighty per cent., were affected. That was a very serious matter if it were a fact. He had given evidence before a Royal Commission on this subject, and if he had thought that fifty per cent. of the animals were affected with the disease he would have hesitated very much in recommending slaughtering and compensation for these animals. (Laughter.) Instead of eighty or fifty per cent., he thought twenty-five would be the utmost percentage of animals infected. He believed that cattle that were brought into cities and put into ill-ventilated byres were most liable to be affected. Where cattle were pastured or kept in well-ventilated byres the outbreak of tuberculosis were few and far between. But when these same animals were brought into the city and tied up in byres with very imperfect ventilation, the disease was very soon transmitted. Tuberculosis was a more infectious disease in cattle than consumption was in the human subject. Professor M'Call mentioned some experiments he had conducted in conjunction with Dr. Carmichael of Glasgow, and proceeding, said that in his opinion scarlatina might be propagated by milk; but whether the organism which produced the disease was received from the cow, or was of a human source he was not prepared to say. If Dr. Russell was correct, it must have originated with the cow.

ASSOCIATION FOR THE STUDY OF COMPARATIVE PSYCHOLOGY IN CONNECTION WITH THE MONTREAL VETERINARY COLLEGE.—A meeting of the above society was held on Monday evening in the Veterinary college, the President, Prof. T. Wesley Mills in the chair.

One of the members, Mr. Skaife, illustrated canine intelligence by demonstrating before the society a number of tricks that he had taught his own dog, a pure bred collie, ten years old. The point of greatest interest was the very short time it had taken to make the dog practically perfect in his performances. The owner of the animal maintained that he understood the meaning of words from their sound independently of gestures or any other accessories, and promised to furnish the society proof of this at a later date. The method of teaching the dog was satisfactorily explained. An account of Dr. Romanes experiments on the sense of smell in dogs, as published in *Nature* for August 21st. 1887, was presented to the society for criticism, also correspondence throwing additional light on this subject. Romanes conclusions from his experiments, which were well performed, are as follows:—

"From the above experiments I conclude that this bitch distinguishes my trail from that of all others by the peculiar smell of my boots, and not by the peculiar smell of my feet. No doubt the smell which she recognizes as belonging distinctively to my trail is communicated to the boots by the exudations from my feet; but these exudations require to be combined with shoe-leather before they are recognized by her. Probably, however, if I had always been accustomed to shoot without boots or stockings, she would have learned to associate with me a trail made by my bare feet. The experiments further show that although a few square millimetres of the surface of one boot is amply sufficient to make a trail which the animal can recognize as mine, the scent is not able to penetrate a single layer of brown paper. Furthermore, it would appear that in following a trail this bitch is ready at any moment to be guided by inference as well as perception, but that the act of inference is instantaneous. Lastly, the experiments show that not only the feet (as these affect the boots) but likewise the whole body of a man exhales a peculiar or individual odor, which a dog can recognise as that of his master amid a crowd of other persons; that the individual quality of this odor can be recognized at great distance to windward, or, in calm weather, at great distance in any direction; and that it does not admit of being overcome by the strong smell of aniseed, or by that of many other footprints."

The President read a letter from a corresponding member in the Canadian Northwest in which was narrated the method by which a young dog had set free his fellow that was chained, by unbuckling the collar. This had been done several times under the observation (unperceived by the dogs) of the correspondent.

A second meeting on Tuesday evening, in the Veterinary college, the President in the chair. After the election of a large number of new members, the experiments of Romanes on the sense of smell in the dog, reported at the last meeting, were discussed; also a communication from Mr. McKechnie, a student of human medicine, relating that a dog had found his way to another at a distance of twelve miles, the only means of his learning of the existence of his fellow being apparently by the sense of smell. This was on the prairie and the only communication between the two places (houses) was by the mail conveyance, which had to cross a creek, so that the scent would, if clinging to the wheels, be immersed in the water before reaching the second dog. Principal McEachran thought it more likely that the scent was detected on the driver, for dogs, etc. were so scarce in prairie homes that they were much appreciated and so greatly fondled.

The Principal also mentioned having himself detected cattle (not visible) on the prairie at a distance of one and a half to two miles, by the sense of smell; that the four horses used by the cowboys always were found together when turned out, though they were not used together. He thought they had a common bond of sympathy from the scent of their master clinging to them; that dog stealers succeeded in their nefarious practices largely from the scent of other dogs attaching to them, especially females, which they had intentionally handled. The sense of smell was so ill-developed in some dogs, especially the stag-hound, that he found it necessary to use a retriever with them when hunting the prairie wolf.

Several members instanced cases of dogs, and even cats, finding their way home, over ways they had not before traveled (or traveled so covered that they could not use the sense of sight) apparently by the sense of smell. The President thought such cases very complex and difficult of explanation, though smell explained some of them no doubt. A communication from a correspondent in Wakefield, Mass., bearing on the sense of smell, etc., of a very profound and subtile nature, was read.

After further remarks by the Hon. President and President, the meeting adjourned.

A third meeting was held in the Veterinary College on Monday evening, the President in the chair. After the consideration of the report of the committee appointed to arrange the details in regard to the granting of a diploma of honorary fellowship, and the election of several new members, Mr. Paige gave a very interesting account of his study of the habits and intelligence of wild bees. He described a series of experimental observations he had made, the conclusions from which were that bees usually take the shortest course home, that they have the sense of smell, and considerable general intelligence.

The President then gave a condensed account of a long series of experiments made upon wasps by two naturalists of Wisconsin. These experiments had proved that wasps have memory, the sense of smell, power to discriminate colors, etc., but were negative as regards hearing, sympathetic emotions, and a special sense of direction. Wasps as well as bees have a high degree of general intelligence, but rank rather lower than bees upon the whole.

A communication to the Society was also read from a veterinary surgeon of Illinois bearing on the intelligence of the dog and the horse. He had been induced to make these communications from reading the President's inaugural address in the JOURNAL OF COMPARATIVE MEDICINE AND SURGERY. The communication made by this correspondent had special reference to the moral nature of dogs and horses, and illustrated the effects of wise treatment and kindness upon a horse thought to be very difficult to manage.

A fourth meeting was held in the Montreal Veterinary College, the President, Dr. T. Wesley Mills, professor of physiology in the McGill University, in the chair. Mr. Skaife, who had spent a considerable portion of a year in studying bee culture on a large bee-farm, described the habits and intelligence of these insects, especially the subject of queen-raising. The necessity of a queen for each hive, the respect paid her, her nuptual flight, young queens, the mailing and shipping for thousands of miles of such, the great fertility of the queen, the remarkable fact that she can lay either only drone eggs or worker eggs, etc., were all described as learned by the actual observation of the reader of the paper. Numerous questions were asked by the members and answered by Mr. Skaife.

The President quoted Sir John Lubbock's opinion that insects (especially bees, wasps, ants, etc.) have the capacity to hear sounds too high in pitch for our ears to appreciate, and to distinguish colors in parts of the spectrum invisible to us. No one had experimented more on bees, wasps and ants than Lubbock. He thought that the greater part of the mental fabric of bees, etc., was to be regarded as of the nature of organized instincts, but not all; and experiments had shown that these instincts were highly plastic. In the light of modern physiology much that was incomprehensible to the older naturalists, in the habits of bees, could now be understood. This was chiefly due to the great and rapid advances in our knowledge of the minute anatomy and the physiology of the nervous system.

Some highly interesting communications from a correspondent in Massachusetts were then read. They seemed to show that a certain Newfoundland dog understood commands given him for the first time, and implied a capacity for interpreting words; but from any point of view the conduct of this dog was puzzling, and quite inexplicable by the views ordinarily entertained of animal intelligence. He warned the members of the danger of interpreting the mental life of the lower animals, especially in groups widely separated from man (insects etc.,) too strictly in accord with man's mental nature. On the other hand, the two could not be wholly different.

A fifth meeting was held in the Veterinary College on Tuesday evening, the President in the chair.

Several communications of much interest were read on the psychic manifestations of dogs from a lady in Massachusetts. One of these described some of the peculiarities of a mongrel dog that had lived with the family his whole life-time of twenty years. He was allowed his own way in everything, like a spoiled child, and showed with much intelligence also many caprices. From the circumstances the study of this dog was valuable. As indicating retentiveness of memory of smells, the case of a setter dog recognizing an old straw hat that had been given away two years before, and picked out by the dog without suggestion from his master, was very striking. Interesting cases of a combination of instinct and general intelligence were cited. Several members discussed the intelligence of the fox, basing their remarks on incidents that had come under their own observation. The fact that foxes can be approached much more readily when man is accompanied by the horse had been observed by several members. The President thought this was to be explained by the law of association, man, when provided with weapons, not being generally on horseback, in this country at least.

Mr. Craig read a paper on a St. Bernard dog, which had been reared at his home. The case illustrated the intelligence of the animal, independent of all training.

A communication was read by the President bearing on the subject of special hereditary instinct and the law of association in the cat. The burying instinct of dogs cats, etc., was discussed. It must be of special importance to animals in a wild state by insuring preservation of food when in excess of present needs.

The final meeting of the Society for the year was held in the Montreal Veterinary College, the President in the chair.

A communication was read from a correspondent in Massachusetts, explaining how a wild cat, when unable to draw a hen through the wire netting of a coop, had dug down beneath the flooring some eighteen inches and then tunnelled along until it came to a hole in the flooring it had seen from without. A remarkable instance of combined memory in a dog was instanced. He had remembered where the blanket he had used to sleep upon in the winter had been put away, and on the first cool night of the following autumn had gone to the drawer in which the blanket had been put and scratched to have it opened, which being done the dog drew out the article and stretched himself upon it after taking it to its former place in the hall of the house. This dog was a brown spaniel with some setter blood in him.

A case illustrating very forcibly how a dog is influenced by the approval or disapproval of his master, and how much more value he sets upon his master than any one of his companions lower in the scale than man, is the following: A large Newfoundland dog had been accustomed to hunt and kill cats which had run wild and become a nuisance. On one occasion he missed catching a cat when sent after her. His master gently reprimanded him. The dog was then missing till morning when he came with a large cat in his mouth dead—one that was a former mess-mate of his. It thus appeared that a cat he must have, even if he had to kill an old friend rather than suffer his master's displeasure.

The President then related an interview he had with an eminently successful horse trainer who had very candidly imparted to him his pro cedure. There were no secrets, and anyone who would pursue these methods with the requisite amount of patience and intelligence must succeed. This trainer concluded that the horse was willing to do what he understood was required of him, and that must be imparted gradually, and with firmness, yet kindness. The horse had a remarkable memory. "He never forgets anything he is once taught thoroughly." People often punish horses for what is traceable directly to their own stupidity. The President believed that the exhibitions of trained animals did good, but might be much more useful if the methods of teaching them were explained, and the public would be content without special sensations.

After discussion of the above subjects, the Society adjourned till next October.

